# Epidemiology and Management of Acute Haematogenous Osteomyelitis in a Tertiary Paediatric Center

**DOI:** 10.3390/ijerph14050477

**Published:** 2017-05-04

**Authors:** Elena Chiappini, Caterina Camposampiero, Simone Lazzeri, Giuseppe Indolfi, Maurizio De Martino, Luisa Galli

**Affiliations:** 1 Paediatric Infectious Disease Unit, Anna Meyer Children’s University Hospital, Florence 50100, Italy; caterina.camposampiero@gmail.com (C.C.); luisa.galli@unifi.it (L.G.); 2 Orthopedics and Traumatology Department, Anna Meyer Children’s University Hospital, Florence 50100, Italy; simone.lazzeri@meyer.it; 3 Department of Pediatrics, Anna Meyer Children’s University Hospital, Florence 50100, Italy; giuseppe.indolfi@meyer.it; 4 Meyer Health Campus, Anna Meyer Children’s University Hospital, Florence 50100, Italy; maurizio.demartino@unifi.it

**Keywords:** acute haematogenous osteomyelitis, children, antibiotic therapy, outcome

## Abstract

*Background*: Paediatric acute hematogenous osteomyelitis (AHOM) is a serious disease requiring early diagnosis and treatment. To review the clinical presentation, management and organisms responsible for AHOM, and to explore risk factors for complicated AHOM, a large cohort referring to a single center over a 6-year period was evaluated. *Methods*: Data from children with AHOM, hospitalized between 2010 and 2015, and aged > 1 month, were retrospectively collected and analyzed. *Results*: 121 children (median age 4.8 years; 55.4% males) were included. Fever at onset was present in 55/121 children (45.5%); the lower limb was most frequently affected (*n* = 68/121; 56.2%). Microbiological diagnosis (by culture and/or polymerase chain reaction (PCR)) was reached in 33.3% cases. Blood and pus/biopsy culture sensitivities were 32.4% and 46.4%, respectively. PCR sensitivity was 3.6% (2/55) on blood, and 66.6% (16/24) on pus/biopsy sample. *Staphylococcus aureus* was the most commonly identified pathogen (*n* = 20); no methicillin-resistant *Staphylococcus aureus* (MRSA) was isolated, 10.0% (*n* = 2) strains were Panton-Valentine-Leukocidin (PVL) producer; 48.8% (59/121) cases were complicated. At univariate analysis, factors associated with complicated AHOM were: recent fever episode, fever at onset, upper limb involvement, white blood count (WBC) ≥ 12,000/µL, C reactive protein (CRP) ≥ 10 mg/L, *S. aureus* infection. At multivariate analyses *S. aureus* infection remained the only risk factor for complicated AHOM (aOR = 3.388 (95%CI: 1.061–10.824); *p*-value = 0.039). *Conclusions*: In this study microbiological diagnosis was obtained in over one third of cases. Empiric treatment targeting methicillin-sensitive *Staphylococcus aureus* seems to be justified by available microbiological data.

## 1. Introduction

Most acute pediatric osteomyelitis are haematogenous infections with an estimated incidence of 8 cases per 100,000 children/year [1,2]. Children below 5 years of age and males are more frequently affected [3]. Early detection of acute hematogenous osteomyelitis (AHOM) is crucial, given that a delay in the diagnosis of only 5 days is a major risk factor for complications [4]. Even if mortality is rare, permanent disabilities can occur, such as growth arrest with limb length discrepancy or deformity [5]. AHOM etiology is changing according to bacterial susceptibility pattern modifications over time, vaccination programs, and implementation of cultural and polymerase chain reaction (PCR) techniques. *Staphylococcus aureus* (*S. aureus*) is the most commonly isolated pathogen [6], accounting for 25–60% of cases of AHOM. Other microorganisms include Group-A *Streptococcus pyogenes*, *Streptococcus pneumoniae*, and Gram-negative rods [5]. *Kingella kingae* is an emergent pathogen [4,7]. Infections sustained by community acquired methicillin resistant *S. aureus* (MRSA) are increasing, particularly in the United States [8]. On the other hand, in Europe community acquired-MRSA is still identified at low rates, while Panton-Valentine-Leukocidin (PVL) producing *S. aureus* isolates are increasingly reported [9]. The empirical therapeutic choice is based on local epidemiological data [4]. An anti-staphylococcal penicillin, i.e., oxacillin or flucloxacillin, or a cephalosporin are generally recommended as first line treatment [4]. Some expert suggest the use of other drugs, including antibiotics effective towards MRSA, depending on local MRSA prevalence [10]. The duration and routes of administration of antibiotics is currently under debate [11]. Historically, AHOM was treated with intravenous (IV) antibiotics for several weeks [12]. More recently, a randomized trial showed that children who displayed a good clinical response after 2–4 days of IV treatment and shifted to oral treatment for further 20 days had not different outcomes when compared to children treated with continued IV therapy for 30 days [13]. A similar approach, using a short IV therapy (about 7 days) has been adopted by several centers in Europe [14] and USA [15], suggesting that short IV therapy may be as effective as longer courses with no increased risk of sequelae. Given the changes in immunization programs, the emergence of MRSA, availability of molecular diagnostics and changing trends in management, a review of the epidemiology of this important disease is needed. Therefore, we evaluated data from a large cohort of otherwise healthy children referring to a single center over a 6-year period.

## 2. Methods

Aim of the present study was to evaluate retrospectively the management and outcome of AHOM in a large single tertiary center over a 6-year period, with particular consideration to possible risk factors for complicated AHOM.

### 2.1. Setting

Meyer Children’s Hospital is a tertiary pediatric university hospital with 200 beds and serves as the main referral center for Tuscany and surrounding regions [16]. The hospital comprises several paediatric departments, including general orthopedic, surgical, intensive care, infectious diseases and other specialist units, working in a multidisciplinary team. Tuscany has a population of three million and six hundred thousand people, 6.2% of the Italian population, with 565,886 people under the age of 18 years [16]. 

### 2.2. Definitions

AHOM was defined as any bone infection presenting with a time period between diagnosis and symptom onset <2 weeks [10,17]. AHOM was diagnosed in the presence of clinical features (fever, swelling, warmth, pain, restriction of movement) and compatible radiologic imaging with or without bacteriological isolation from blood or bone sample [14]. 

### 2.3. Complicated Osteomyelitis

AHOM was defined as complicated when the child developed sepsis, septic shock, or arthritis, cellulitis, sub-periosteal or muscle abscess, deep venous thrombosis (DVP) [14], pathologic fracture, septic emboli or when the child necessitated admission to the intensive care unit (ICU) [4].

### 2.4. Study Design and Population

A retrospective single center study was conducted, evaluating data from all children aged between 1 month and 18 years, consecutively admitted to Meyer Children’s Hospital with a discharge code consistent with the diagnosis of osteomyelitis (ICD codes M86.00-86.99) , according to the World Health Organization International Classification of Diseases (WHO ICD-10), between 1 January 2010 and 31 December 2015. Data from these children were independently reviewed by two authors. Inclusion criteria was AHOM, as defined above, and exclusion criteria were: age ≤ 30 days; congenital or acquired immunodeficiency, underlying bone disease; carrier of prosthetic materials; hospital acquired infection, previous history of skin wound, open fracture or surgery at the site of bone infection or in contiguous areas, septic arthritis (i.e., no adjacent osteomyelitis on imaging). Only cases fulfilling the above criteria were included in the study. Data were collected from medical records, electronic records for laboratory, and radiology results. Demographic and clinical details, microbiological and radiologic results, and clinical management including type, route and duration of antibiotic treatment, need for surgery, and readmission to hospital within 6 months of initial diagnosis were entered into an electronic database. Reasons for changes in the antibiotic regimen were also recorded (i.e., clinical and/or radiological failure; switch from IV to oral therapy; drug toxicity; switch from empirical therapy to a targeted antimicrobial therapy after bacterial isolation).

Surgery was performed in children not responding to medical treatment, or needing draining an abscess or in children with adjacent arthritis needing draining and joint lavage.

### 2.5. Laboratory and Microbiological Investigations

All the laboratory tests were performed in the same laboratory at the Author’s Institution using standardized techniques and according to manufacturer’s instructions. Hematological parameters (WBC, CRP, ESR) were collected at admission before administration of any antimicrobial therapy. In particular CRP in serum or heparinized plasma was detected by means of particle enhanced immunonephelometry on the Dimension Vista™ System equipped with the Dimension Vista™ System Flex^®^ reagent cartridge (Siemens Healthcare Diagnostics, Marburg, Germany). The Analytical Measurement Range (AMR) is 0.29–19.0 mg/dL. ESR was measured using a capillary micro-photometer method, and expressed in mm/hour. The AMR is 2–120 mm/h.

Blood cultures and cultures from bone or joint fluid samples were processed using standard methods. All samples were processed for detection of common human bacteria (or cultured for detection of MRSA isolates) using rich and selective culture media. After 48 hours of incubation in aerobic atmosphere at 37 °C, plates were read in order to detect the presence of pathogens. Identification of bacteria isolates was based on typical colony morphology on selective culture media (Mannitol Salt 2 Agar, Mac Conkey Agar, bioMérieux, (Geneva, Switzerland)) or on chromogenic culture media (chromID^®^ CARBA SMART, bioMérieux and BBL™ CHROMagar™ MRSA II, Becton Dickinson, (Becton Dickinson, Lincoln Park, NJ, USA). In order to confirm species-level identification mass spectrometry analysis was performed using MALDI-TOF (VITEK^®^ MS, bioMérieux). Organisms were identified phenotypically and confirmed using traditional methods or the Vitek2 gram positive card (bioMérieux). Antibiotic susceptibility testing was performed using an automated system (Vitek2 AST-P612 card, bioMérieux) and the Kirby–Bauer disk diffusion method in accordance with the guidelines of the Clinical and Laboratory Standards Institute. Antibiotic susceptibility was evaluated using the automated system VITEK^®^2 (bioMérieux) with the card AST-P632 for the several antibiotics. The European committee on antimicrobial susceptibility testing (EUCAST) clinical breakpoints were used as interpretation criteria. The pathogenicity of *S. aureus* infections is related to various bacterial surface components and to extracellular proteins such as PVL. In order to determine the potential virulence of MRSA strains, a specific PCR assay for the presence of the gene (lukS-lukF) encoding for the PVL was set up following a previously published protocol [18]. 

Universal real-time PCR assay targeting the gene coding for 16S ribosome RNA coupled with sequencing of amplified products was performed on blood and/or tissue samples, as previously described [19,20]. 

### 2.6. Statistical Methods

Data were reported as median and interquartile range (IQR) or absolute numbers and percentages. Non parametric Mann-Whitney test, Fisher’s exact test or Chi Square test were used to compare continuous or categorical variables, as appropriate. All significant tests were two-sided. Uni- and multi-variate logistic regression analyses were performed to investigate the association between several parameters and risk of complicated AHOM, calculating odds ratios (ORs) and 95% confidence intervals (CIs). Variables included in univariate analyses were gender, age, site of infection (upper limb, lower limb, other site), previous trauma, previous fever episode, clinical characteristics at the time of admission, WBC (≥ or <12,000 cell/µL) CRP (≥ or <10 mg/L), ESR (≥ or <20 mm/h); initial X-ray (positive or negative); initial ultrasound (US) (positive or negative); initial MRI (positive or negative); positive microbiological culture or PCR on blood or pus; infection sustained by *S. aureus* vs. other/unknown; first antibiotic treatment duration (≥ or <10 days) and type of first antibiotic regimen. Factors significantly associated to complicated AHOM at univariate analysis were included in the multivariate analyses. All statistical analyses were carried out using the SPSS (Statistical Package for the Social Sciences, SSPS Inc., Chicago, IL, USA) for Windows software program version 19.0. A *p*-value < 0.05 was considered significant. The study was approved by the Ethics Committee at the authors’ institution (121/2016). 

## 3. Results

Overall, 121 children were included in the study (Table 1). Median age was 4.8 years (IQR: 1.6–10.5); 67/121 (55.4%) children were males; 117/121 (96.7%) children were Caucasian; 2/121 (1.6%) children were Asian and 2/121 (1.6%) were Hispanic. Thirteen out of 121 (10.7%) children were immigrants (born abroad). None of the children had a positive history for recent travel. Previous trauma (within 15 days) was reported in 25/121 children (20.7%). Ten children participated in sport activities (8.3%); 66/121 (54.5%) had a recent episode of fever. Median interval between symptom onset and hospital admission was 6 days (IQR: 2–12) and 60 children (49.6%) were admitted 5 days after symptom onset. Fever at presentation was present in 55/121 children (45.5%) while 117/121 (96.7%) displayed at least one inflammation sign. In particular, 67/121 children (55.4%) presented with swelling, 58/121 (47.9%) with increased warmth, 111/121 (91.7%) with pain and 45/121 (37.2%) with erythema at the affected site; 97/121 (80.2%) had functional limitation.

The lower limb was most frequently affected (*n* = 68/121; 56.2%), followed by the upper limb (*n* = 21/121; 17.4%). Localization was on long bones in 68/121 (56.2%) of cases. The metaphysis was involved in 43 cases (68%), the diaphysis in 6 (8.8%). Extension of the infection from the metaphysis to the diaphysis was observed in 19 cases (27.9%), while an epiphyseal involvement was observed in 12/68 (17.6%). The pelvis was involved in 15 cases (12.3%) and there were seven discitis (5.7%). 

Fifty-nine children out of 121 (48.8%) had a complicated AHOM. In particular, 4/121 (3.3%) children developed deep vein thrombosis (DVT); 22/121 (18.2%) sepsis/septic shock, 32/121 (26.4%) arthritis, 20/121 (16.6%) cellulitis, 16/121 (13.2%) sub-periosteal abscess, 13/121 (10.7%) muscle abscess, 5/121 (4.13%) pathologic fracture, 2/121 (1.7%) septic emboli and 7/121(5.8%) needed to be admitted to ICU. 

With respect to uncomplicated cases, complicated cases more frequently involved the upper limb (27.1% vs. 8.1%; *p*-value = 0.005) and more frequently reported a recent episode of fever (69.5% vs. 40.3%; *p*-value = 0.001). We observed a higher proportion of complications when long bone were involved vs. non long bone (*p*-value = 0.021). Children with complicated AHOM presented more frequently fever at onset (59.3% vs. 32.3%; *p*-value = 0.002) (Table 1). Other factors associated with complications were, WBC ≥ 12,000/µL, CRP ≥ 10 mg/L, a positive microbiological test, infection sustained by *S. aureus* and a positive MRI. In the multivariate analyses only infection sustained by *S. aureus* was associated with risk of complicated AHOM (OR = 3.388 (1.061–10.824); *p*-value = 0.039) (Table 2).

### 3.1. Inflammatory Indices

WBC < 12,000/µL was detected in 68/115 (59.1%) patients; 28/68 (41.2%) were complicated and 40/68 (58.8%) uncomplicated children (*p*-value = 0.030). ESR <20 mm/h was observed in 27/96 (28.1%) children; 15/27 (55.6%) were complicated and 12/27 (44.4%) uncomplicated (*p*-value = 0.495). CRP < 10 mg/L was observed in 39/113 (34.5%) children; 11/39 (28.2%) were complicated and 28/39 (71.8%) uncomplicated (*p*-value < 0.001). 

### 3.2. Microbiological Tests

Overall, 72/121 (59.5%) children underwent at least one microbiological test (cultures or PCR assay on blood or bone sample): a pathogen was isolated in 24/72 cases (33.3%). 

A blood culture was performed in 37/121 children (30.6%) and it resulted positive in 12/37 cases (32.4%). A culture of the pus/biopsy was done in 28/121 children (23.1%) and a pathogen was isolated in 13/28 of them (46.4%). PCR assay on blood samples was performed in 55/121 (45.5%) cases and was positive only twice (3.6%). PCR assay on pus/biopsy samples was performed in 24/121 (19.8%) children and was positive in 16/24 (64.0%). In complicated cases the number of positive cultures/PCR was significantly higher than in uncomplicated cases (complicated 21/27 (77.8%); vs. uncomplicated 6/27 (22.2%); *p*-value < 0.001). 

*S. aureus* was by far the most common observed pathogen, detected with culture in 20/72 (27.8%) tested children. It was isolated from 11/15 (73.3%) positive blood cultures, 10/13 (76.9%) positive pus/biopsy cultures, 1/2 (50.0%) positive PCR on a blood sample and 12/16 (75.0%) positive PCR on a pus/biopsy sample. Among those tested, 17/43 (39.5%) of complicated AHOM and 3/29 (10.3%) of uncomplicated were positive for *S. aureus* respectively (*p* = 0.006) Two out of 20 (10.0%) *S. aureus* strains were positive for PVL, and all (20/20; 100%) were methicillin-sensitive. There was only one isolate with aminoglycoside resistance but susceptibilities to clindamycin, levofloxacin, and trimethoprim-sulphametoxazole (TMP-SMX) were preserved along with oxacillin susceptibility. Combined clindamycin and erythromycin resistance was reported in three isolates that maintained susceptibilities to oxacillin, TMP-SMX, aminoglycosides and levofloxacin. One isolate showed resistance to TMP-SMX, levofloxacin and erythromycin while maintaining oxacillin and clindamycin susceptibility.

Other organisms isolated were: *Streptococcus pyogenes* in 1/15 (6.7%) blood culture and 1/13 (7.7%) cultures on pus and 2/16 (12.5%) PCR on a pus sample; *Proteus mirabilis* in 1/13 (7.7%) culture on pus, *Pseudomonas aeruginosa* in 1/13 (7.7%) pus cultures, *Streptococcus agalactiae* in 1/2 (50.0%) PCR on a blood sample, *Fusobacterium necrophorum* in 1/16 (6.3%) PCR on a pus sample and *Streptococcus pneumoniae* in 1/16 (6.3%) PCR on a pus sample (Table 3). No *Kingella kingae* isolate was found.

The cases with PVL-positive *S. aureus* infection were two females, aged 3 and 9 years, who developed AHOM complicated by sub-periosteal abscess in one case and necrotizing pneumoniae and septic shock, admission to ICU, in the other. Both patients required prolonged IV therapy with second-line antibiotics and surgery. The older child developed a sequela (angular deformity).

### 3.3. Imaging Studies

Conventional radiography was performed in 102/121 patients (84.3%). The diagnostic yield of conventional radiography was low (*n* = 7/102; 6.9%). US was performed in 64/121 children (52.9%), and displayed a compatible image only in 5/64 children (7.8%). MRI was performed in 111/121 cases (91.7%) and findings supportive of OM were evidenced in 104/111 cases (93.7%). CT was marginally used (10/121 children (8.3%)) and findings supportive of OM were present in 6/10 (60.0%). Bone scintigraphy was performed in 5/121 children (4.1%), with supportive findings of OM in all cases.

### 3.4. Medical and Surgical Treatment

All the 121 children received IV antibiotics at admission (Table 3). The most frequently administered antibiotic regimen was the combination oxacillin plus one cephalosporin (91/121; 75.2%), followed by a glycopeptide based therapy (*n* = 12/121; 9.9%) and a clindamycin based therapy (*n* = 5/121; 4.1%). 

In 64/121 (52.9%) the first therapy was switched to a second IV antibiotic regimen for the following reasons: clinical failure (9/64 children; 14.1%), targeted therapy after pathogen identification (12/64; 18.8%), allergic reaction (6/64; 9.4%), simplification from a double therapy to a single-drug therapy (28/64; 43.8%). Reason was not specified in 9 cases (14.1%) 

Twenty-two out of 27 (77.8%) children with at least a positive microbiological test switched to a second IV treatment; 18/22 (81.8%) with complicated AHOM and 4/22 (18.2%) with uncomplicated AHOM (*p*-value = 0.303). Seven out of 22 (31.8%), all complicated, switched to a second IV therapy for a clinical failure; 9/22 (40.9%) switched to a target therapy after receiving result of microbiological tests, 1/22 (4.5%) passed to a second IV therapy for allergic reaction and 5/22 (22.7%) switched for therapeutic simplification.

Eighty-eight out of 121 (72.7%) children switched to an oral antibiotic therapy; most commonly amoxicillin/clavulanic (69/88; 78.4%). Other used oral antibiotics were clindamycin (*n* = 4), ciprofloxacin (*n* = 3), linezolid (*n* = 3). Overall 20/121 (16.5%) children underwent surgery, 15/20 (75.0%) with a complicated osteomyelitis and 5/20 (15.0%) with a uncomplicated (*p*-value: 0.002); 7/121 (5.8%) children underwent surgery for two times.

### 3.5. Follow-Up Results

9/121 (7.4%) children were switched to a second IV therapy for clinical failure with subsequent complete resolution; 4 children out of 121 (3.3%) were hospitalized twice for clinical relapse. Median follow-up of the study children was 6.7 months (IQR 3.3–8.9 months). Two children (1.6%) developed mild sequelae (angular deformity). 

## 4. Discussion

Availability of local data is important in order to optimize local therapeutic protocols [10]. To our knowledge this is one of the largest studies on paediatric AHOM performed in Europe. Similar cohort studies have been previously conducted in Spain [14], and France [21], as well as in other extra-European countries including Israel [22], Thailand [23], Australia [3], and the United States [24]. 

Results from these studies highlight differences in local microbiological data which may be, at least partly, influenced not only by geographical variations, but also by the availability of sophisticated microbiological/PCR assays. As an example, the reported prevalence of *Kingella kingae* infection ranges from 0% up to 82% children with osteo-articular infection [25,26], probably because its identification is challenging and requires targeted aerobic blood culture vials or real-time PCR technique. 

Our study confirms epidemiological data previously well described in literature [5]: AHOM is more commonly reported in males and in young children, it more frequently involves the lower limb and long bones, and the patient’s history is commonly positive for a recent trauma or a febrile episode. In our dataset fever at admission was present only in 45% of children. This finding is in accordance with that one reported by Dartnell et al. [17] who observed fever as the presenting symptom only in 61.7% of children, while pain and swelling and erythema where present in 81.1% and 70% of the cases, respectively. We also comfirmed that WBC has a low sensitivity compared to ESR and CRP. Similarly, in other studies, leukocytosis has been reported only in 36% of children, but increased ESR was observed in 91% of children and CRP in 81% of them [27]. As expected, we observed a low sensitivity of X-ray imaging [28] and US [4]. 

Microbiological identification of the causative pathogen in our dataset was consistent with previous results [14]: a microorganism was identified bin more than one third of cases. The sensitivity of blood and pus culture was 32.4% and 46.4%, respectively. The sensitivity of the PCR assay was only 3.6% on blood sample and reached 64.0% on pus/biopsy sample, underlying the importance of performing PCR assay on pus/biopsy specimens. 

*S. aureus* was the most commonly identified etiological pathogen, but no MRSA strain was isolated. This result is in contrast with data reported from the United States [8,29] but is consistent with other European findings [14,16,17,30]. It should be noticed that, in our dataset 10.0% *S. aureus* strains were PVL producers. Both cases were complicated. In Europe PVL producing *S. aureus*, even if most commonly methicillin sensitive, has been associated with more severe infections, therefore treatment should be aggressive and should include an antibiotic with antitoxin effect (i.e., clindamycin, linezolid, or rifampicin) [9,31]. We observed that commonly prescribed antibiotics in our dataset included glycopeptides, carbapenems, or quinolones or a double therapy with cephalosporin plus one penicillin. Since these regimens are not recommended as first line treatment for AHOM in children, interventions aimed to promote a more appropriate use of antibiotics in our setting are needed.

Our study has several limitations, due to its retrospective nature. Moreover, only 59.5% of the children underwent at least one microbiological investigation before commencing an antibiotic therapy and follow-up was limited to 6 months. Another limitation is that although PCR16S is commonly used in our Center to identify bacterial infection, a specific real time PCR for *K. kingae* is not available. This may have led to an underestimation of *K. kingae* infections. 

## 5. Conclusions

Complications occur frequently in children with AHOM and are strongly related to *S. aureus* infection. In this study microbiological diagnosis was obtained in over one third of cases. Empiric treatment targeting methicillin-sensitive *Staphylococcus aureus* seems to be justified by available microbiological data.

## Figures and Tables

**Table 1:** Characteristics of the 121 study children admitted to a single tertiary Center with acute osteomyelitis (AHOM).

Characteristics		Total	Not-Complicated AHOM 62 (51.2%)	Complicated AHOM 59 (48.8%)	*p*-Value
Sex (*n*; %)	Female	54	30 (48.4%)	24 (40.7%)	0.393
	Male	67	32 (51.6%)	35 (59.3%)	
Ethnicity (*n*; %)	Caucasian	117	59 (95.2%)	58 (98.3%)	0.619 (vs. others)
	Asian	2	2 (3.2%)	0 (0.0%)	
	Hispanic	2	1 (1.6%)	1 (1.7%)	
Median age (months; IQR)			59 (21–125)	58 (17–130)	0.669
Interval between symptom onset and diagnosis (days; IQR)			5 (2–11)	7 (5–10)	0.671
Median length of total antibiotic therapy (IV plus oral) (days; IQR)			35 (30–39)	41 (31–48)	0.037
Recent travel (*n*; %)	Yes	0	0 (0.0%)	0 (0.0%)	
Recent Trauma (*n*; %)	Yes	25 (20.7%)	10 (16.1%)	15 (25.4%)	0.206
Involved bone (Grouped Site) (*n*; %)	Upper limb	21 (17.4%)	5 (8.1%)	16 (27.1%)	0.008
	Lower limb	68 (56.2%)	35 (56.5%)	33 (55.9%)	0.954
	Other	32 (26.4%)	22 (35.5%)	10 (16.9%)	0.021
Site (*n*; %)	Humerus	13 (10.7%)	3 (4.9%)	10 (16.9%)	0.040
	Ulna	3 (2.5%)	0 (0.0%)	3 (5.1%)	
	Radius	1 (0.8%)	0 (0.0%)	1 (1.7%)	
	Clavicle	2 (1.7%)	1 (1.6%)	1 (1.7%)	
	Scapula	1 (0.8%)	0 (0.0%)	1 (1.7%)	
	Carpal bones	1 (0.8%)	1 (1.6%)	0 (0.0%)	
	Phalanges of the hand	2 (1.7%)	1 (1.6%)	1 (1.7%)	
	Mandible	1 (0.8%)	1 (1.6%)	0 (0.0%)	
	Skull bones	1 (0.8%)	1 (1.6%)	0 (0.0%)	
	Ribs	2 (1./%)	2 (3.2%)	0 (0.0%)	
	Vertebrae	11 (9.1%)	9/62 (14.5%)	2 (3.4%)	
	Hip	16 (13.2%)	9 (14.5%)	7 (11.9%)	
	Femur	19 (15.7%)	5 (8.1%)	14 (23.7%)	0.023
	Patella	1 (0.8%)	0 (0.0%)	1 (1.7%)	
	Tibia	17 (14.0%)	8 (12.9%)	9 (15.2%)	
	Fibula	3 (2.5%)	1 (1.6%)	2 (3.4%)	
	Tarsus bone	23 (19.0%)	17 (27.4%)	6 (10.2%)	0.015
	Metatarsus and phalanges of the foot	6 (5.0%)	5 (8.1%)	1 (1.7%)	
Multifocal AHOM (*n*; %)	Yes	6 (5.0%)	3 (4.8%)	3 (5.1%)	1.000
Sport (*n*; %)	Yes	10 (8.3%)	1 (1.6%)	9 (15.3%)	0.007
Recent episode of fever (*n*; %)	Yes	66 (54.5%)	25 (40.3%)	41 (69.5%)	0.001
Fever at onset (*n*; %)	Yes	55 (45.5%)	20 (32.3%)	35 (59.3%)	0.002
At least an inflammation sign at onset (*n*; %)	Yes	117 (96.7%)	59 (95.2%)	58 (98.3%)	0.619
Swelling (*n*; %)	Yes	67 (55.4%)	29 (46.8%)	38 (54.4%)	0.051
Warmth (*n*; %)	Yes	58 (47.9%)	23 (37.1%)	35 (59.3%)	0.014
Pain (*n*; %)	Yes	111 (91.7%)	54 (87.1%)	57 (96.6%)	0.095
Redness (*n*; %)	Yes	45 (37.2%)	16 (25.8%)	29 (49.2%)	0.007
Biopsy executed (*n*; %)	Yes	22	11 (16.1%)	11 (18.6%)	0.745
Positive blood PCR (*n*; %)	Yes	3/55 (5.5%)	1/21 (4.8%)	2/34 (5.9%)	1
Positive pus/biopsy PCR (*n*; %)	Yes	17/25 (68.0%)	5/7 (71.4%)	12/18 (66.7%)	1
Positive blood culture (*n*; %)	Yes	15/37 (40.5%)	1/15 (6.7%)	14/15 (93.3%)	0.108
*Staphylococcus aureus* positivity (*n*; %)	Yes	20/72 (27.8%)	3/29 (10.3%)	17/43 (39.5%)	0.006

Note: AHOM: acute hematogenous osteomyelitis.

**Table 2:** Factors associated with complicated acute osteomyelitis at uni- and multi-variate analyses.

Factors	*n*/N	Univariate Analysis OR (95%CI)	*p*-Value	Multivariate Analysis	*p*-Value
				aOR (95%CI)	
Recent fever					
Yes	41/66 (62.1%)	2.197 (1.058–4.564)	0.035	1.541 (0.641–3.707)	0.334
Fever at onset					
Yes	35/55 (63.6%)	2.308 (1.111–4.795)	0.025	1.086 (0.439–2.687)	0.859
Grouped Site					
Lower limb/other	43/100 (48.5%)	1		1	
Upper limb	16/21 (76.2%)	2.444 (1.909–6.573)	0.007	1.7 (0.565–5.111)	0.345
WBC ≥ 12,000/µL					
Yes	29/47 (61.7%)	2.363 (1.117–4.998)	0.024	1.873 (0.801–4.379)	0.148
CRP ≥ 10 mg/L					
Yes	47/74 (63.5%)	2.774 (1.234–6.238)	0.014	1.771 (0.718–4.370)	0.215
*Staphylococcus aureus*					
Yes	17/20 (85.0%)	4.614 (1.577–13.504)	0.005	3.388 (1.061–10.824)	0.039

Note: factors not significantly associated with complication at univariate analysis were: gender, age < 3 years, presence of swelling, warmth, pain, redness, ESR > 20 mm/h; IV therapy < 10 days; type of antibiotic regimen.

**Table 3:** Antibiotic therapy in complicated and uncomplicated acute osteomyelitis.

Antibiotic Therapy	Complicated Acute Osteomyelitis (*n* = 59)	Uncomplicated Acute Osteomyelitis (*n* = 62)
IV therapy; days (median and IQR)	20 (12–24)	20 (12–23)
Oral therapy; days (median and IQR)	13 (4–15)	13 (1–14)
Total treatment; days (median and IQR)	36 (30.5–44.5)	36 (30–44.75)
IV Drugs		
Single therapy	0	3
Combination therapy	59	59
Oral therapy		
Single therapy	44	36
Combination therapy	5	4
